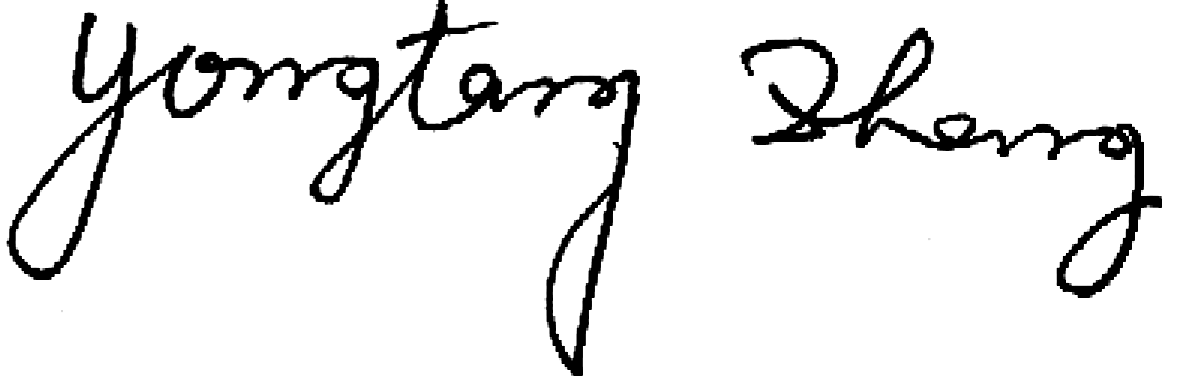# An“impact”in publishing

**DOI:** 10.24272/j.issn.2095-8137.2019.040

**Published:** 2019-07-18

**Authors:** Yong-Gang Yao, Yun Zhang, Yong-Tang Zheng

**Affiliations:** 1 Kunming Institute of Zoology, Chinese Academy of Sciences, Kunming Yunnan 650223, China

## Abstract

On 20 June 2019, Clarivate Analytics (2019) announced its Journal Citation Reports of 2018. From this, *Zoological Research* (*ZR*) received its first impact factor based on citations in 2018 for indexed papers published during 2016 to 2017. Although the new impact factor (1.556) is modest, it ranks *ZR* at 52 among the 170 SCI journals (quartile 2) in the Zoology category. This excellent result is not only a reflection of your enduring support, but also in recognition of our efforts to boost *ZR* from a Chinese-language only journal in 1980 to an English-language only journal of international standing by 2014.

From its original founding by a group of first-generation zoologists in “New China”, *ZR* has aimed to publish the latest advances and scientific findings in the field of zoology. This goal has remained unchanged for the past four decades and has allowed *ZR* to become a respected platform that encourages fair and open scientific discussion. We hope that the rigorous and meaningful science published in *ZR* will reach ever wider audiences as such outstanding research deserves international recognition. It is for this reason that we continue to publish via open access.

With the official announcement of our latest impact factor, we have witnessed a boost in submissions from researchers across the world within the zoological field. In addition to the recently announced impact factor, we also believe this increase to be a reflection of our reputation as a prompt and well-respected platform for the dissemination of cutting-edge and high-quality research. As such, we wish to emphasize the journal’s unique areas of interest and warmly welcome submissions with a research focus on: (1) Primates and Animal Models; (2) Conservation and Utilization of Animal Resources, and (3) Animal Diversity and Evolution.

*ZR* also continues to maintain its academic publishing integrity and reinforce its publishing policies (Editorial Office of Zoological Research, 2016; Liu, 2016; Yao & Jiang, 2018). Most recently, *ZR* amended the ethics requirements regarding submissions involving field animal surveys and sample collection, as well as animal material import and export, which applies to all submissions since 01 June 2019. From that date, all submissions must clearly state whether “*all applicable international, national, and/or institutional guidelines for the care and use of animals were strictly followed; all animal sample collection protocols complied with the current laws of XX (country name)*…” or not; and “*permission/document/series numbers for conducting scientific field surveys and/or material transfer agreements*” must be provided (Editorial Office of Zoological Research, 2019).

In addition, for papers that have encountered problems with previous peer review, *ZR* offers a chance for successful and fast publication for those authors whose work is of a suitably high standard. Authors can submit their manuscript along with a point-by-point reply to the issues and comments raised by the reviewers. Such submissions will be fairly and promptly evaluated by *ZR* editorial board members and experts in the field, and, if accepted, be guaranteed fast-track publication.

Peer review is an effective and vital measure to ensure the academic quality of publications. However, the improper handling of conflicts of interest (COIs) during manuscript review can introduce bias in evaluating research findings. Thus, *ZR* authors must disclose all potential COIs and all reviewers and editors must follow the recommendations of the International Committee of Medical Journal Editors (ICMJE) in handling potential COIs. Reviewers and editors must declare all COIs relevant to the work under consideration (i.e., relationships, both financial and personal, that may interfere with the interpretation of the work) to avoid potential bias and, furthermore, must not use knowledge of the work they are reviewing before its publication in furtherance of their own interests (International Committee of Medical Journal Editors, 2017, 2018).

Receiving its first impact factor is another stepping-stone along the evolutionary path of *ZR*. With strong support from our editors, reviewers, authors, and readers, *ZR* will continue to progress and improve. We hope that the pride we feel in publishing your excellent research is shared in return. Should you have any exciting work or constructive suggestions you would like to share, please do not hesitate to contact us.

**Figure:**
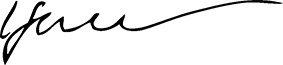


**Figure:**
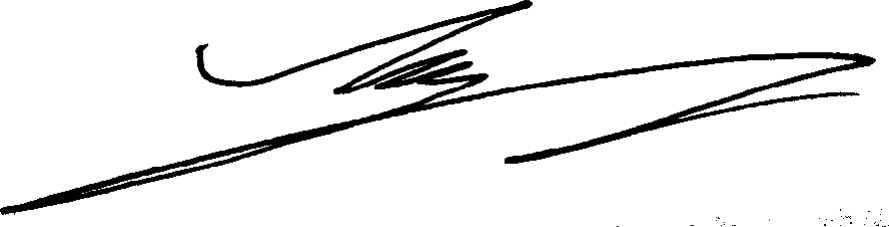


**Figure:**